# What is hip preservation?

**DOI:** 10.1093/jhps/hny015

**Published:** 2018-05-16

**Authors:** Richard (Ricky) Villar

**Affiliations:** Editor-in-Chief, Journal of Hip Preservation Surgery

What do we mean when we use the word ‘preservation’? Are we conserving the whole joint, just the bone, perhaps only the articular cartilage? Or, are we setting the scene for repair, or are we simply preserving function? Therein lies a problem, I sense, as when it comes to preservation, each of us means something different. One could say that preservation is synonymous with hip arthroscopy, but clearly it is not. One could say that any surgery to the hip that does not involve arthroplasty could be defined as a preservation manoeuvre. After all, the United Kingdom has something called the Non-Arthroplasty Hip Register. And yet, surely even arthroplasty surgeons are preserving the hip in some way?

How about resurfacing, interposition, stubby stems, bipolar replacements, cementless joints and even hemiarthroplasties? Are these not preserving portions of the hip to some degree, in case something further is needed? Look at our forefathers, who undertook arthrodesis, osteotomy, hanging hips and plenty more. In their way they were trying to preserve natural tissue. Preservation as a concept, and as a technique, has been around for decades, probably centuries in reality. We are not as new as we like to think. All surgeons preserve something when they operate, just look at the fixation of fractures.

Indeed, when it comes to preservation, must we physically operate at all? After all, even the management of femoral neck osteoporosis is preserving the hip in its way. And believe me, in my humanitarian life, when as a surgeon I am patching up war wounded, replacing the hip of a 25-year-old who has been shot through the pelvis, the femoral head now being non-existent, is itself a form of preservation.

Way back, when this journal was first created, as Editor-in-Chief I received a letter from a surgeon asking if she might submit a paper on the late effects of resurfacing. At that time, I declined the suggestion. I had in my mind that any form of arthroplasty meant the hip was no longer preserved. Yet look at us now, burring away cam lesions, taking down rims, removing, yes removing articular cartilage and sometimes excising whole labra in order to reconstruct. Anatomy is removed, sometimes small, sometimes large, so that something further may be created. There are so many definitions when it comes to preservation. What appears without doubt is that conserving hip function if far more multidisciplinary than we might have originally thought.

So, from there arises my suggestion, an idea which I pray creates debate. Should this journal, our journal, not open its doors wider and encompass the full spectrum of hip preservation? Assuming we agree that each of us sees preservation differently, why not encourage submissions from physicians, whose aim is to keep patients from us? Why not welcome our arthroplasty colleagues provided they seek to preserve the hip in some way? Does it truly matter that a portion of metal is placed within a joint? It is what the practitioner has sought to preserve that counts, not necessarily the way it has been achieved.

My comments will be like a red flag to a bull for some, food for thought to others, but do send me your ideas should you have the opportunity. As an editorial team we manifestly have views but let us hear yours as well.

Turning to the last issue of *JHPS*, again we were spoiled for choice. The moment I feel I am up to speed with the globe’s involvement in hip preservation, in comes a submission that makes me realize I am actually out of date.

Our last issue, number 5.1, had a fair few papers that encouraged any of us who may still not be repairing labra to think again. There was the paper by Carton and Filon [1] on preserving the chondrolabral interface, that by Locks *et al.* [2] on the outcomes after labral repair with capsule or rectus femoris, or that by Weidner, Wyatt and Beck [3] on labral augmentation with the ligamentum teres. Labral repair is in, it seems that labral resection is out.

Meanwhile this issue, issue 5.2, excites me enormously. Have a look at the content once you have accessed the issue and you will see how, perhaps for the first time, there is much more to our content than just hip arthroscopic surgery. There is plenty on venous thromboembolism after hip preservation surgery [4], be that open or arthroscopic [5]. I am relieved the authors concluded that the prevalence of venous thromboembolism after hip preservation surgery is, as they describe it, ‘acceptably low’. There is plenty on other things, too.

There is one metric, if that is the right description, that also excites me and that is our most frequently read paper. It shows how the hip preservation community is thinking. First prize was for a long time held by John O’Donnell and his unit’s excellent review of the ligamentum teres [6]. Now, step forward Hal Martin and his team whose 2015 paper on the deep gluteal syndrome [7] has moved upward to pole position. It is telling, too, that none of our top three papers has anything to do with femoroacetabular impingement. My how times have changed.

So, as ever, please enjoy this issue of *JHPS*. It is published for you, the hip preservation practitioner, and is filled from cover to cover with brilliance. I commend this issue to you in its entirety.

My very best wishes to you all.
